# A Revised Taxonomy of the *Bassia scoparia* Complex (Camphorosmoideae, Amaranthaceae s.l.) with an Updated Distribution of *B. indica* in the Mediterranean Region

**DOI:** 10.3390/plants14030398

**Published:** 2025-01-28

**Authors:** Alexander P. Sukhorukov, Zhibin Wen, Anastasiya A. Krinitsina, Alina V. Fedorova, Filip Verloove, Maria Kushunina, Jean-François Léger, Mathieu Chambouleyron, Abbès Tanji, Alexander N. Sennikov

**Affiliations:** 1 Department of Higher Plants, Biological Faculty, Lomonosov Moscow State University, 119234 Moscow, Russia; ankrina@gmail.com; 2 State Key Laboratory of Desert and Oasis Ecology, Key Laboratory of Ecological Safety and Sustainable Development in Arid Land, Xinjiang Institute of Ecology and Geography, Chinese Academy of Sciences, Urumqi 830011, China; zhibinwen@ms.xjb.ac.cn; 3 Xinjiang Key Laboratory of Conservation and Utilization of Plant Gene Resources, Urumqi 830011, China; 4 The Specimen Museum of Xinjiang Institute of Ecology and Geography, Chinese Academy of Sciences, Urumqi 830011, China; 5 Tsitsin Main Botanical Garden, Russian Academy of Sciences, 127276 Moscow, Russia; alina_77777@mail.ru; 6 Meise Botanic Garden, Nieuwelaan 38, 1860 Meise, Belgium; filip.verloove@plantentuinmeise.be; 7 Department of Plant Physiology, Biological Faculty, Lomonosov Moscow State University, 119234 Moscow, Russia; mkushunina@gmail.com; 8 Reneco International Wildlife Consultants LLC, 39th Floor, Sky Tower, Reem Island, Abu Dhabi P.O. Box 61741, United Arab Emirates; jfleger@reneco.org; 9 Reneco North Africa, Rue de Midelt, Rabat 10020, Morocco; mchambouleyron@reneco.org; 10 Independent Researcher, Settat BP 589, Morocco; abbestanji1@gmail.com; 11 Botanical Museum, Finnish Museum of Natural History, University of Helsinki, 00100 Helsinki, Finland; alexander.sennikov@helsinki.fi

**Keywords:** Amaranthaceae, *Bassia*, classification, distribution, plant invasion, Mediterranean, molecular phylogeny, taxonomy

## Abstract

*Bassia scoparia* is a widespread weedy species in the temperate regions of the world and is valued as a medicinal and ornamental plant. To date, the taxonomic concept of *B. scoparia* remains insufficiently studied due to a limited number of samples used in the previous phylogenetic analyses. To solve the taxonomy of the *B. scoparia* complex, we constructed a new phylogeny based on the nuclear ribosomal internal transcribed spacer (ITS), plastid intergenic spacer *atpB-rbcL,* and plastid region *rpL16* intron sequences for numerous samples with diverse morphology. Our analysis revealed a close proximity and intermixed positions of the samples of the *B. scoparia* group with various morphology. Because of this polyphyly, we prefer to broadly delimit the species. An updated nomenclature of *B. scoparia* is provided including four new synonyms: *Bassia angustifolia*, *B. littorea*, *Kochia albovillosa,* and *K. scoparia* subsp. *hirsutissima*. In its new circumscription, *B. scoparia* encompasses populations with glabrous or variously hairy leaves and perianths. The original material of *Kochia sieversiana*, previously considered a species with hairy leaves and inflorescences, has the same diagnostic characters as in *B. scoparia* s.str. The correct name for more hairy-leaved plants is *B. scoparia* var. *subvillosa.* Plants with hairy perianths known as *Kochia albovillosa* and *K. scoparia* subsp. *hirsutissima* have a restricted distribution in Central Asia and South Siberia and have never been recorded as alien in other regions; they can be classified as a separate variety, *B. scoparia* var. *hirsutissima*. The ornamental variant of oblong or pyramidal shape may be called *B. scoparia* var. *trichophila*. *Bassia scoparia* is often confused with a similarly looking relative, *B. indica*, especially in North Africa, a region where secondary ranges of both species overlap. Phylogenetically, these species are sister groups; they share some morphological characters but have different primary distribution ranges. We traced a recent expansion of *B. indica* in the Mediterranean with the first record reported from the European continent (Spain) and uncovered various introduction pathways of the species in this region.

## 1. Introduction

*Bassia scoparia* (L.) Voss (formerly *Kochia scoparia* L.) is widespread through the temperate and subtropical regions of the world, but its exact origin in Eurasia still remains uncertain [1]. It is easily recognized in the field due to its annual life form, mostly bushy habit, flat leaves with a petiole-like base, leafy inflorescences, and perianths usually having tubercles or short wings at the fruiting stage. In other characters like leaf shape, the presence of tufts of hairs at the base of leaf-like bracts bearing flower clusters, and the shape of perianth outgrowths (if present), *B. scoparia* is extremely variable [2,3]. This fact encouraged many authors of major floristic treatments to accept some segregate species in the previously widely used genus *Kochia* Roth, e.g., *K. sieversiana* (Pall.) C.A.Mey. [4,5,6], *K. densiflora* Aellen, and *K. angustifolia* (Turcz.) Peschkova [7], all known in Siberia and subsequently adopted in Russian herbaria and local floras and checklists. In addition, Sukhorukov [8] described *K. scoparia* subsp. *hirsutissima* Sukhor. from Central Asia, characterized by its hirsute stem, leaves, and perianth segments. Such plants appeared to be similar to *K. albovillosa* Kitagawa, a forgotten species described from northeastern China [9].

Recently, *Kochia* (type species: *K. arenaria* (Maerkl.) Roth = *K. laniflora* (S.G.Gmel.) Borbás) was reduced to a synonym of *Bassia* All. based on its molecular phylogeny [10]. A major part of the *Kochia* species was transferred to *Bassia*, or their position was confirmed in the latter genus from the morphological point of view (e.g., [11,12,13,14]). The *Bassia scoparia* clade, the topic of our study, appeared in this phylogeny as a separate group, corresponding to the traditional *B.* sect. *Semibassia* G.Beck [11] (p. 155).

Among other species accepted in *Bassia*, Kadereit and Freitag [10] proposed two new combinations in the *B. scoparia* clade: *B. angustifolia* (Turcz.) Freitag & G.Kadereit (≡ *Kochia scoparia* var. *angustifolia* Turcz.) from South Siberia and *B. littorea* (Makino) Freitag & G.Kadereit (≡*Kochia littorea* (Makino) Makino) from Japan; the latter species was not included in the phylogenetic analysis and was transferred to *Bassia* mostly based on the zig-zag inflorescences. Moreover, both species seem to differ from *B. scoparia* by their narrow and rather thick leaves having several layers of water storage tissue in the mesophyll, an adaptation to the saline substrates on which they occur [15]. The other species in the *B. scoparia* group were considered synonyms [10]. The phylogenetic group of *B. scoparia + B. angustifolia* was found to be a sister to *B. indica* (Wight) A.J.Scott, another species that is morphologically very close to the *B. scoparia* group and is distinguished by its regularly pubescent perianth segments. In contrast to *B. angustifolia*, both *B. scoparia* and *B. indica* are typical ruderal plants in their secondary ranges (e.g., [1,4,16,17,18]).

The *B. scoparia* group remains poorly understood. Sukhorukov [1] provided a list of synonyms for this species, but some further species and infraspecific taxa described in this group have not been evaluated properly, including *B. angustifolia* that was considered to be restricted to the southern parts of Central and Eastern Siberia [5,7,19]. Due to the limited phylogenetic and morphological sampling of the *B. scoparia* alliance, used in the phylogenetic study by Kadereit and Freitag [10], the number of species in this group still remains uncertain.

To resolve this issue, we have examined additional samples of *B. scoparia* s.l. taken from recently collected herbarium specimens with various morphological characters. This analysis allows us to reconsider the species rank for the taxa described in this group, and to establish a revised infraspecific classification. Following the re-circumscription of *B. scoparia*, we have re-evaluated its morphological differences from *B. indica*, a close relative of *B. scoparia* that is often confused with the latter species. Distributional ranges of both species have been verified, and the Mediterranean distribution of *B. indica* has been updated on the basis of herbarium specimens and original observations.

## 2. Results

### 2.1. Phylogenetic Study of Bassia scoparia

The concatenated matrix (supermatrix) contains 91 taxa (including five outgroups) and 2226 characters from three molecular loci: one nuclear (ITS) and two plastid (*atpB-rbcL* intergeneric spacer and *rpL16* intron). The aligned ITS region contains 642 characters, while the *atpB-rbcL* intergeneric spacer and *rpL16* intron contain 736 and 848 characters, respectively. The total number of variable characters in all partitions is 397 (i.e., 18% of the characters in the supermatrix are variable). The IQ-Tree software version 2 [20] found the best tree (−log likelihood 6876.1912) using the TIM3e+I+G4 model for ITS partition, and K3Pu+F+R2 or G4 models for two plastid partitions (*atpB-rbcL* intergeneric spacer and *rpL16* intron), respectively.

**Figure 1:**
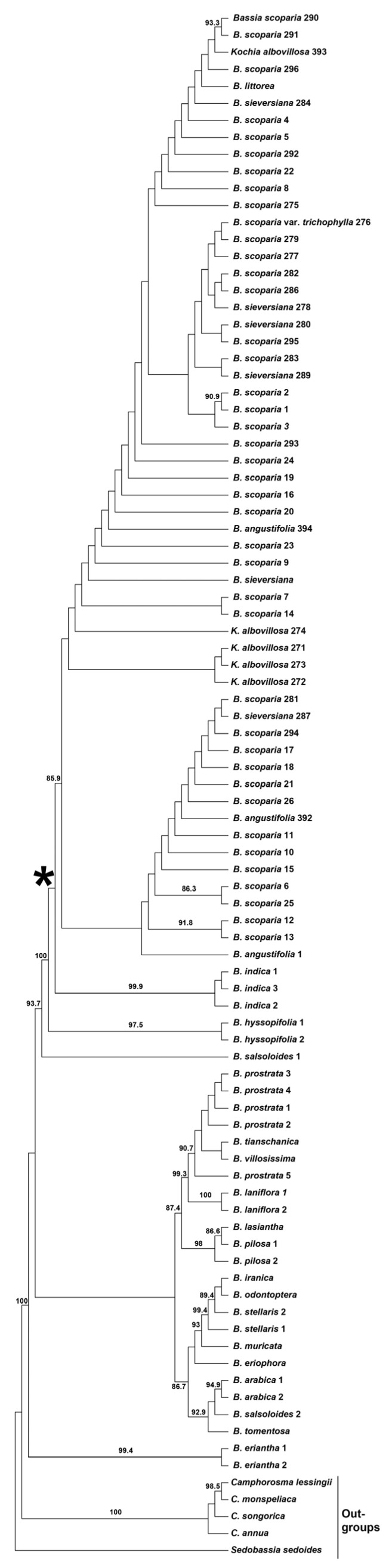
Phylogeny of *Bassia* and outgroups, recovered from the partitioned Maximum Likelihood (ML) analysis of the concatenated dataset (ITS, *atpB-rbcL* intergeneric spacer, and *rpL16* intron) and presented as a cladogram. Numbers above or below branches indicate ML aLRT support values [21]. The figure shows only clades that received aLRT support higher than 0.8. An asterisk denotes the clade corresponding to the *B. scoparia* taxonomic alliance.

The most important results of this study are as follows:

(1) *Bassia angustifolia* (three accessions), *B. littorea* (one accession)*, B. scoparia* s. str. (incl. *B. scoparia* var. *trichophila*) (41 accessions), *B. sieversiana* (six accessions), and *Kochia albovillosa* + *K. scoparia* subsp. *hirsutissima* (five accessions) are all nested in the moderately supported (aLTR = 80.8) *Bassia scoparia* clade (Figure 1) that strictly corresponds to the *B. scoparia* taxonomic alliance. Neither of these segregate species (including narrowly defined *B. scoparia*) appears monophyletic.
(2) The single accession of *B. littorea* is defined as a non-supported sister of a clade containing three accessions of *B. scoparia* (290, 291, and 296) and one accession of *K. albovillosa* (393).
(3) The latter taxon is monophyletic with the exclusion of accession 393. However, the clade with four samples of *K. albovillosa* (271–274) received no aLRT support.
(4) The monophyletic *B. indica* is confirmed as a moderately supported sister of broadly defined *B. scoparia* (aLTR = 100 and 80.8) (Figure 1).
(5) The monophyletic *B. hyssopifolia* is a strongly supported sister of the clade (*B. scoparia* s.l. plus *B. indica*) (aLTR = 100 and 98) (Figure 1).
(6) The short lengths of all branches of the obtained ML phylogenetic tree within the *B. scoparia* clade (Figure S1) imply a high degree of genetic similarity among the analyzed individuals from all the species listed above.

### 2.2. Revised Taxonomy and Nomenclature of Bassia scoparia

Based on the constructed phylogenetic tree, the merger of *B. angustifolia* and *B. littorea* with *B. scoparia* has been confirmed. In its expanded circumscription, *B. scoparia* includes many synonyms, which are comprehensively listed below. Typifications have been traced and, when necessary, updated.

***Bassia scoparia*** (L.) Voss, Der Deutsche Gartenrat 2 (132. Extra-Beilage): [1] (1904); Beck in Reichenbach, Icon. Pl. Germ. Helv. 24: 155 (1909), isonym; A.J.Scott, Feddes Repert. 89(2–3): 108 (1978), isonym;

≡*Chenopodium scoparia* L., Sp. Pl.: 221 (1753);

≡*Atriplex scoparia* (L.) Crantz, Inst. Rei Herb. 1: 208 (1766);

≡*Kochia scoparia* (L.) Schrad., Neues J. Bot. 3(3–4): 85 (1809);

≡*Salsola scoparia* (L.) M.Bieb., Mém. Soc. Imp. Naturalistes Moscou 1: 144 (1811);

≡*Bushiola scoparia* (L.) Nieuw., Amer. Midl. Naturalist 4: 95 (1915).

Lectotype (Jafri & Rateeb in Jafri & El-Gadi, Fl. Libya 58: 26 (1978)): Herb. Linnaeus 313.20 (LINN—image seen!). Image available at: http://linnean-online.org/3145/ (accessed on 26 July 2024).

Note: Plants with ciliate leaves, inconspicuous tufts of hairs in the bract axils, and glabrous perianths.

=*Suaeda sieversiana* Pall., Ill. Pl.: 45 (1803);

≡*Kochia sieversiana* (Pall.) C.A.Mey. in Ledebour, Fl. Altaic. 1: 415 (1829);

≡*Kochia scoparia* [var.] *subglabra* Moq., Chenop. Monogr. Enum.: 91 (1840);

≡*Kochia scoparia* [var.] *soongorica* Moq. in Candolle, Prodr. 13 (2): 131 (1849), nom. illeg. superfl.;

≡*Kochia scoparia* var. *sieversiana* (Pall.) Ulbr. in Ascherson & Graebner, Syn. Mitteleur. Fl. 5(1): 163 (1913);

≡*Kochia scoparia* f. *subglabra* (Moq.) Aellen, Mitt. Basler Bot. Ges. 2(1): 15 (1954);

≡*Bassia sieversiana* (Pall.) W.A.Weber, Phytologia 67: 426 (1989).

Lectotype (designated by Brignone & Denham, Ann. Missouri Bot. Gard. 106: 16 (2021)): Ill. Pl., Table 38 (1803).

Note: Plants with ciliate leaves, scant tufts of hairs in the bract axils, and ciliate perianths.

=*Kochia scoparia* [var.] *subvillosa* Moq., Chenop. Monogr. Enum.: 91 (1840);

≡*Kochia scoparia* [var.] *densiflora* Moq. in Candolle, Prodr. 13(2): 131 (1849), nom. illeg. superfl.;

≡*Kochia densiflora* B.D.Jacks., Ind. Kew. 2(1): 10 (1895); Aellen, Mitt. Basler Bot. Ges. 2(1): 13 (1954), isonym;

≡*Kochia scoparia* subsp. *densiflora* (B.D.Jacks.) Aellen in Hegi, Ill. Fl. Mitteleur., ed. 2, 3(2): 710 (1961); Aellen, Mitt. Basler Bot. Ges. 2(1): 15 (1954), comb. inval. provis;

=*Bassia scoparia* subsp. *densiflora* (B.D.Jacks.) Cirujano & Velayos, Anales Jard. Bot. Madrid 44(2): 577 (1987), comb. inval.;

≡*Bassia scoparia* var. *subvillosa* (Moq.) Lambinon, Bull. Soc. Échange Pl. Vasc. Eur. Occid. Bassin Médit. 26: 33 (2000).

Holotype: [Russia, Republic of Buryatia] In hortis oleraceis ad stationem Lipow, prope Kiachtam, 1829, Turcz.[aninow] 171 (G-DC, isotype LE!).

Note: A variety with ciliate leaves and prominent tufts of hairs in the bract axils. An isotype at LE was erroneously designated as a lectotype by Sukhorukov [1], p. 308.

=*Kochia scoparia* [var.] *angustifolia* Turcz., Bull. Soc. Imp. Naturalistes Moscou 25(4): 424 (1852);

≡*Kochia angustifolia* (Turcz.) Peschkova, Stepnaya Fl. Baikal’skoi Sibiri: 53 (1972);

≡*Bassia angustifolia* (Turcz.) Freitag & G.Kadereit, Taxon 60(1): 73 (2011).

Lectotype (designated here): [Russia] In salsis Dauriae, 1831, Turcz.[aninow] (LE01286677!).

Note: *Bassia angustifolia* represents smaller plants of *B. scoparia* with narrow (linear or lanceolate) leaves growing on saline substrates.

=*Kochia scoparia* [var.] *chinensis* Turcz., Bull. Soc. Imp. Naturalistes Moscou 25(4): 424 (1852).

Described from cultivation (North China origin). Type probably at LE (not found).

*=Bassia scoparia* var. *culta* Voss [publication place unknown];

≡*Kochia scoparia* var. *culta* (Voss) Probst, Mitt. Naturf. Ges. Solothurn 6: 26 (1920); Farwell, Pap. Michigan Acad. Sci. 26: 10 (1940), isonym;

≡*Kochia scoparia* subsp. *culta* (Voss) O.Bolòs & Vigo, Butll. Inst. Catalana Hist. Nat., Secc. Bot. 38(1): 88 (1974);

≡*Bassia scoparia* subsp. *culta* (Voss) Nebot, De la Torre, Mateo & Alcaraz, Anales Biol., Fac. Biol., Univ. Murcia 16: 104 (1990).

Described from cultivation. Lectotype (designated here): [icon] “Besen-Strandhaar” in Der Deutsche Gartenrat 2 (132. Extra-Beilage): [1] (1904).

=*Kochia trichophila* Stapf ex “Haage & Schmidt”, Möllers Deutsche Gärtn.-Zeitung 21(18): 219 (1906);

≡*Kochia scoparia* var. *trichophila* (Stapf ex “Haage & Schmidt”) Osborn, Gardeners’ Chronicle, ser. 3, 39: 167 (1906);

≡*Kochia scoparia* f. *trichophila* (Stapf ex “Haage & Schmidt”) Schinz & Thell., Mitt. Bot. Mus. Univ. Zürich 46: 10 (1909);

≡*Bassia scoparia* f. *trichophila* (Stapf ex “Haage & Schmidt”) S.L.Welsh, Utah Fl., ed. 3: 113 (2003).

Described from cultivation. Neotype (designated here): [icon] Curtis’s Botanical Magazine 145: Table 8808 (1919).

Note: A cultivated ± glabrous form with a bushy habit and narrowly lanceolate or linear leaves.

=*Kochia scoparia* var. *littorea* Makino, Bot. Mag. Tokyo 23: 12 (1909);

≡*Kochia littorea* (Makino) Makino, Bot. Mag. Tokyo 27: 254 (1913);

≡*Kochia scoparia* f. *littorea* (Makino) Kitam., Acta Phytotax. Geobot. 20: 206 (1962).

Lectotype (designated here): Japan, Suruga prov. [Honshu Island, Shizuoka pref.] Shimidzu [town], T. Makino (MAK4173!).

Note: A variety with rather thick and almost glabrous leaves and slightly curved inflorescence, known from the coastal areas of Japan and the Korean Peninsula.

=*Kochia alata* Bates, Amer. Bot. (Binghamton) 24: 52 (1918);

≡*Bassia alata* (Bates) A.J.Scott, Feddes Repert. 89(2–3): 108 (1978).

Lectotype (designated by Brignone & Denham, Ann. Missouri Bot. Gard. 106: 16 (2021)): USA, Nebraska, Adams Co., Hastings, 2 October 1917, J. Bates 6607 (US00102665; isolectotype NY01185891!).

Note: An image kept at NY shows the plants with winged perianths. This taxon was described from North America, where *B. scoparia* is an alien species.

=*Kochia scoparia* var. *alata* C.H.Blom, Acta Horti Gothob. 3: 154 (1927).

Holotype: China. Hubei: “Hsiao-wu-tai-shan [39.853° N, 114.986° E], inter Sin-pao-an et Hun-ho, in arenosis, ca. 600 m”, 14 August 1921, Harry Smith 255 (GB).

Note: Plants with winged perianths, independently described from China.

=*Kochia albovillosa* Kitag., Rep. Exped. Manchoukuo, sect. IV, part 4, Index Fl. Jeholensis: 78 (1936).

Holotype: [China, Heilongjiang prov.] Hsing-an occid., in pratis siccis arenosis circa O-nyû-to, 27 September 1929, T. Nakai, M. Honda & H. Kitagawa 734 (TI00010552!).

Note: A specimen with rather thick leaves, woolly flower clusters, hirsute perianths (glabrescent at fruiting), and segments with unequal wings. Later, Kitagawa [22] incorrectly sunk it to the synonymy of *Kochia sieversiana*.

=*Kochia scoparia* var. *suaedifolia* Kitag., Liniamenta Fl. Mansh.: 191 (1939), as ‘*suaedaefolia*’;

*≡Kochia scoparia* f. *suaedifolia* (Kitag.) K.P.Ma, Fl. Heilongiangensis 4: 404 (1992).

Holotype: [China, Liaoning prov.] Feng Tien [Fengtian] prov., in arenosis prope Cheng-chia-tun, 21 August 1931, M. Kitagawa s.n. (TI? n.v.).

Note: A psammophytic variety with fleshy subulate leaves [22]. This variety is probably found in saline habitats and may represent the plants similar to *B. scoparia* var. *angustifolia*.

=*Kochia parodii* Aellen, Verh. Naturf. Ges. Basel 50: 151 (1939).

Lectotype (designated by Brignone & Denham, Ann. Missouri Bot. Gard. 106: 16 (2021)): France, Grand Est, Haut-Rhin, Mulhouse, Wollkompost der Firma Lädrich, 17 August 1938, P. Aellen s.n. (US00344773, image seen!; isolectotypes A00037204, F0054127F, MW0591998!).

Note: The specimens in US and MW are represented by vegetative twigs typical of *B. scoparia* var. *trichophylla*.

=*Kochia parodii* var. *elongata* Aellen, Darwiniana 5: 121 (1941).

Lectotype (designated by Brignone & Denham, Ann. Missouri Bot. Gard. 106: 16 (2021)): Argentina, Región de Bahía Blanca, Punta Alta, March 1930, J.F. Molfino s.n. (BA8813).

Note: A variant of *B. scoparia* with elongated inflorescences [23].

=*Kochia parodii* var. *densa* Aellen, Darwiniana 5: 122 (1941);

≡*Kochia scoparia* f. *densa* (Aellen) A.Soriano, Revista Argent. Agron. 12: 54 (1945).

Lectotype (designated by Brignone & Denham, Ann. Missouri Bot. Gard. 106: 16 (2021)): Argentina, Buenos Aires, Pdo. Trenque Lauquen, Trenque Lauquen, Laguna El Hinojo, 14 March 1938, A.L. Cabrera 4336 (LP013669).

Note: A variant of *B. scoparia* with compact inflorescences [23].

=*Kochia parodii* var. *glabrescens* Aellen, Darwiniana 5: 122 (1941).

Lectotype (designated by Brignone & Denham, Ann. Missouri Bot. Gard. 106: 16 (2021)): Argentina, Buenos Aires, Pdo. Trenque Lauquen, Laguna El Hinojo, 14 March 1938, A.L. Cabrera 4337 (LP013668).

Note: A typical form of *B. scoparia*, described anew from its secondary distribution area.

=*Kochia scoparia* var. *appendiculata* Parsa, Kew Bull. 3(2): 226 (1948);

≡*Kochia scoparia* f. *appendiculata* (Parsa) Aellen, Mitt. Basler Bot. Ges. 2: 15 (1954).

Holotype: Iran. Kerman, in incultis, 1900 m, 23 August 1892, J. Bornmüller 4220 (K000898796!; isotype HBG-524770, image seen!).

Note: A plant with subglabrous leaves and short-winged perianths at the fruiting stage.

=*Kochia scoparia* f. *subscoparia* Aellen, Mitt. Basler Bot. Ges. 2: 16 (1954).

Holotype: Iran, bei Mahmudieh nördl.[ich von] Teheran, verlassenes Schweinezucht-Terrain, 1948, P. Aellen 549 (G).

Note: A form hardly distinguishable from *B. scoparia* var. *trichophila*.

=*Kochia sicorica* O.Bolòs & Masclans, Butll. Inst. Catalana Hist. Nat. 38: 89. (1974);

≡*Bassia sicorica* (O.Bolòs & Masclans) Greuter & Burdet, Willdenowia 13: 282. (1984).

Holotype: [Spain, Catalonia] Almacelles, in Salsolo-Peganie, 3 May 1958, F. Masclans s.n. (BC579242).

Note: A tiny plant with an unbranched stem (n.v., according to Bolòs & Masclans in [24]).

=*Kochia scoparia* subsp. *hirsutissima* Sukhor., Ann. Naturhist. Mus. Wien 104B: 700 (2003).

Holotype: [East Kazakhstan], planities demissa Balchasch-Alakulensis, in regione cursus medii fl. Lepsy et lac. Baskan-Kul, ripa boreali-occidentalis lac. Baskan-Kul, pratum salsum, 30 June 1934, I.A. Linczevsky & O.A. Linczevsky 263 (LE!).

Note: Plants with hirsute leaves and perianths from Central Asia and North China.

### 2.3. Updated Diagnostic Characters and Distribution of Bassia indica in the Mediterranean

***Bassia indica*** (Wight) A.J.Scott, Feddes Repert. 89: 108 (1978);

*≡Kochia indica* Wight, Icon. Pl. Ind. Orient. 5: t. 1791 (1852);

*≡Kochia scoparia* subsp. *indica* (Wight) Aellen, Mitt. Basler Bot. Ges. 2: 15 (1954).

Lectotype (designated by Sukhorukov, PhytoKeys 116: 109 (2019)): [India, Tamil Nadu state] Coimbatore, March 1847, Wight 2479 (K000400258!).

*Bassia indica* is a sister clade to the *B. scoparia* group [10]. We confirm its current phylogenetic position (Figure 1) using an additional sample from Spain (see Table S1), where it has been recently found.

The phylogenetic proximity of *B. indica* and *B. scoparia* is not surprising because both species share broad leaves and winged or tuberculate perianths at the fruiting stage. Although *B. indica* is morphologically always characterized by a tall bushy habit (tumbleweed), thick hirsute leaves, and villous perianths, these characters may also be present in some forms of *B. scoparia* growing in Central Asia and South Siberia, which are known as *Kochia albovillosa* and *K. scoparia* subsp. *hirsutissima*. Thus, *B. scoparia* and *B. indica* have partly overlapping morphological traits (see Table 1). Nevertheless, in the regions where both species have overlapping secondary ranges, e.g., in Europe, West Asia, and North Africa, they are clearly well distinguishable from each other [25], including branching pattern, leaf recurvation, and the pubescence of the perianths. Furthermore, the plants of *B. scoparia* with glabrous or hirsute perianths do not have recurved leaves and do not form a true tumbleweed habit.

Compared to *B. scoparia*, which occurs in the temperate climate and is occasionally found in the subtropics (e.g., in North Africa), the native distribution range of *B. indica*, which was described from southern India [26], encompasses the tropical and subtropical regions of Asia.

To date, the secondary range of *B. indica*, which is native to the Indian subcontinent, encompasses huge territories including the Flora Iranica area [27,28], Arabian Peninsula [29,30], South Africa [31], East Tropical Africa [32], Mozambique [33], Sudan [34], and many countries of the Mediterranean Region.

According to the Euro+Med Plantbase [35], *B. indica* is known in the Mediterranean area from Cyprus, Egypt, Israel/Palestine, Jordan, and Libya. These data are generally based on the references for Libya [36], Egypt [37], Israel/Palestine ([38], as *Kochia indica*), Cyprus (erroneously recorded as ‘native’; see [39]), and Jordan [40].

Its spread in the Mediterranean Region was said to be connected with the cultivation of *B. indica* in Egypt as a green summer fodder plant in 1930s ([41], as *Kochia indica*). The first collection in Egypt was in late 1920s from Alexandria City [17]. Nevertheless, the Egyptian ‘origin’ of the secondary distribution of *B. indica* is not an initial stage of its spread in adjacent countries. For instance, the species was already known from the territory of historical Palestine in the late 1910s when it was described from Yaffa City (now territory of Israel) as *B. joppensis* Bornm. & Dinsm. [42], and was considered alien there [43]. We conclude that the spread of *B. indica* into the Mediterranean Region may have occurred independently in two waves: (1) unintentionally from West Asia and (2) from cultivation in Egypt (see also remarks below about *B. indica* in Morocco).

**Figure 2:**
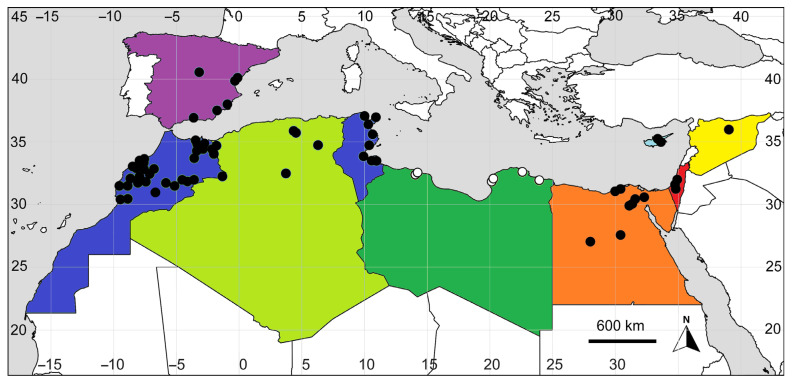
Distribution of *Bassia indica* in the Mediterranean. Each color indicates first records, namely, red 1910+ (Israel/Palestine), orange 1930+ (Egypt), yellow 1950+ (Syria), bright green 1960+ (Algeria), dark green 1970+ (Libya), light blue 1990+ (Cyprus), dark blue 2000+ (Morocco, Tunisia), and violet 2020+ (Spain). Black dots indicate records based on the herbarium specimens, empty dots indicate records taken from [36].

Below, we summarize and critically evaluate our knowledge on the distribution of *B. indica* in some Mediterranean countries (cited from east to west, see also Figure 2; for the specimens seen in the herbaria visited, see Appendix B).

Israel: First collected in late 1910s (described as *B. joppensis*).

Egypt: *Bassia indica* was used as a fodder plant in 1930s; at present, it is considered as one of the most impactful invasive plants in the country [44].

Syria: Known from the 1950s (G!, see Appendix B).

Cyprus: *Bassia indica* has been known in Cyprus (which is part of the European Union but, biogeographically, rather belongs to Asia) for several decades [39,45,46,47,48].

Libya: The first collections of *B. indica* are from the mid-1970s [36], with further spread in various parts of the country [49]. It is reported as a dominating species in some degraded areas [50]. Surprisingly, it is not recognized as an invasive species in Libya [51].

Tunisia: *Bassia indica* was reported as a new invasive species in Tunisia by Sukhorukov et al. [32] based on the absence of the species in [52]. Despite the lack of previously published records, it had been already collected from Djerba by J. Lambinon in 2005: Gouvern. de Medenine, île de Djerba, côte NE, plage de la Seguia, Ras Tourgueness, talus ruderalisé en haut de pré salé, 5 January 2005, *J. Lambinon* 05/Tu/32 (LG; dupl. BR0000005027378: https://www.botanicalcollections.be/specimen/BR0000005027378, accessed on 27 March 2024).

Algeria: *Bassia indica* is widespread at least in North Algeria, based on Benmeddour and Fenni ([53], with references therein), but they erroneously named it *Kochia scoparia*. Quézel and Santa ([54], as *Kochia indica*) mentioned that the species has been cultivated as a forage plant. We were unable to see any specimens from Algeria. Nevertheless, based on [53], the species seems to be invasive in the country and not a casual alien as reported earlier [55].

Morocco: A report by Zahran ([56], as *Kochia indica*) of the presence of the species in Morocco, based on [57] (cited by Zahran as “Dahandiez & Marie”), is erroneous. *Bassia indica* was indeed collected for the first time in Morocco in 2004 [58], near Guercif and Taourirt towns in the northeast, and then by Alain Dobignard (https://www.floramaroccana.fr/bassia-indica-cle.html, accessed on 15 November 2024) close to Marrakech. It was more recently reported again from the north-eastern part of the country, near Jerada town [59]. Since these first dates, the species has rapidly spread in all the semi-arid and arid areas of the country, where it is now especially abundant along roadsides and other disturbed places (cultivated fields, wastelands, etc.).

It seems that the previously cultivated plants called *Kochia scoparia* in Tanji and Taleb [60], which invade the fields, in most cases belong to *B. indica*. Some authors of the present paper (MC, APS) have seen *B. scoparia* as an ergasiophyte on the regularly irrigated fields only in a few localities of Morocco. On the contrary, *B. indica* is commonly found in abandoned fields in many parts of Morocco. If our assumption about a misidentification of both species is correct, the first introduction of *B. indica* in the country would date from 1948, when seeds were brought for forage research in experiment stations at Oujda and Rabat [60].

Herein, we also report the first documented records of *B. indica* from continental Europe.

**Figure 3:**
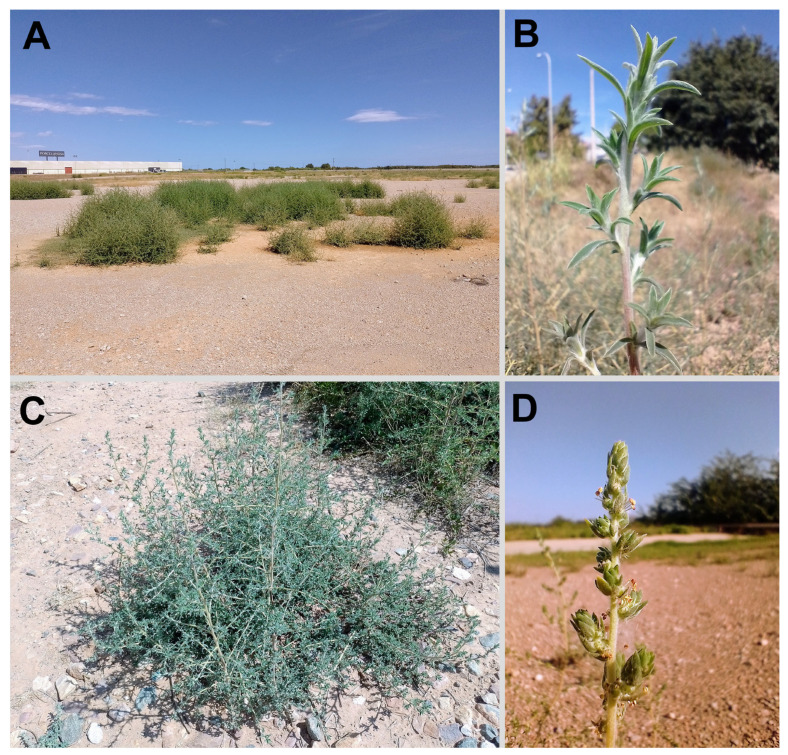
*Bassia indica* in Spain. (**A**) *B. indica* in Vila-real, 29 August 2023. The species has a typical tumbleweed habit. (**B**) The typical dense stem indumentum of *B. indica* (Vila-real, 30 August 2023). (**C**) *B. indica* on bare, sunlit, and stony ground at the railway station of Vila-real, 30 August 2023. (**D**) Inflorescence of *B. indica* in Vila-real, 11 September 2023. Photographs by F. Verloove.

Spain: province of Castellón: Vila-real (Villarreal), Camí del Cabeçol next to Riu Anna, bare steppe-like area, very common, 29 August 2023 (flowers: 11 September 2023), *F. Verloove* 14892 (BR0000027058565V); Burriana, Avinguda de l’Unió Europea, rough ground in residential area, roadsides, etc., common, 30 August 2023, *F. Verloove* 14897 (BR0000027058480V) (Figure 3).

During fieldwork, focused on research into urban and other highly anthropogenic habitats in the wider Castellón area (province of Castellón, Spain), a huge population with at least 1000 individuals of *B. indica* was discovered on 29 August 2023. The plants were observed in an expansion area of the local industrial zone in a levelled steppe-like landscape next to the Anna River in Vila-real. It was subsequently also seen in numerous other localities in the same area, to such an extent that its presence was only recorded during three days of fieldwork, i.e., between 29th and 31th of August (the species was so widespread and clearly present for a long time that it was impossible to document all sites). In addition to the initially detected locality, it was also observed in Alquerías del Niño Perdido (roadsides and along tracks in orchards next to the Anna River; also, as an urban weed near the railway station), Burriana (vacant lots and roadsides in recent residential development; also, at the estuary of the Millars River), Vila-real (along railway embankments), Nules (roadsides and rough ground near the beach), and Moncofa (disturbed ground in the urban area).

In the Castellón area, *B. indica* has been observed in an area that covers ca. 150–200 km^2^. It is represented by thousands of individuals and either is a fast-spreading recent introduction or—perhaps more likely—it has been overlooked for quite a long time, doubtlessly due to its morphological resemblance to *B. scoparia* (see above). A revision of all relevant herbaria (especially MA) was not possible in the context of this study, but no specimens were found in BC (Barcelona), which may indicate that the species is missing from the (climatologically less favorable) northeastern part of the Iberian Peninsula. However, a cursory check of some online observation platforms (iNaturalist, observation.org) showed that the species also occurs unnoticed elsewhere in Spain. Its presence was detected in the provinces of Alicante (Pilar de la Horadada), Almería (El Calón, San Juan de los Terreros), Granada (Salobreña), and Guadalajara (Pozo de Guadalajara), all—except for the latter, which is at more or less the same latitude as Castellón—located in the southern half of the Iberian Peninsula.

In the recently detected Spanish populations, the species was observed to start flowering 2–3 weeks later than *B. scoparia*, although it is unclear whether this can be generalized.

## 3. Discussion

### 3.1. Taxonomic Circumscription of Bassia scoparia

Our phylogenetic results (Figure 1 and Figure S1) are consistent with morphological observations. Segregate taxa were distinguished within the *B. scoparia* taxonomic alliance mainly on the basis of the presence and density of pubescence of the leaves, bracts, and perianths, as well as subtle differences in the lamina shape. Morphologically weakly differentiated taxa (*B. angustifolia*, *B. littorea*, *B. sieversiana*, and *K. albovillosa*), which were accepted as species by the past authors, all fall into a large and undifferentiated clade (Figure 1), which corresponds to the broadly defined *B. scoparia*. Thus, we find it most reasonable to consider the latter species in its broad circumscription, including taxonomically insignificant forms (previously treated as “species”) with different variants of pubescence and details of leaf (lamina) shape.

The re-circumscription of species limits in the *Bassia scoparia* complex has triggered the question of its native distribution area and the means of its secondary dispersal. Although the origin of *Bassia scoparia* has not been confirmed yet, it may be traced to Central Asia and South Siberia, where the greatest diversity of morphological forms (described as *Kochia angustifolia*, *K. sieversiana*, *K. albovillosa,* and *K. scoparia* subsp. *hirsutissima*) are present. Sukhorukov [8] indicated that the species is alien at least in Europe and West Kazakhstan, and this conclusion has been recently confirmed by Sukhorukov et al. [61] for Orenburg Region of Russia, which is situated in the easternmost part of Europe.

### 3.2. Valid Publication of Bassia scoparia

Nursery catalogues and horticultural periodicals belong to the “grey” taxonomic literature. Such publications may be ephemeral in terms of preservation, i.e., unlikely to survive in public libraries or private collections, and obscure in terms of knowledge circulation, i.e., possibly well known to the contemporaneous public but obsolete to modern botanists. In many cases, new plant names have been overlooked in such literature [62].

The existence of obscure historical publications may be completely neglected, to the extent that no apparent traces can be found even in contemporaneous references [63]. Even in modern times, overlooked publications may contain extensive masses of new species names, disturbing the established nomenclature and threatening its stability [64]. On the other hand, beneficial effect of old horticultural publications has also been observed due to their early reports of rare cultivation [62].

*Bassia scoparia* has been a popular ornamental plant since the end of the 19th century [65,66,67,68], although its garden use can be traced back to the 18th century [69]. It is no wonder that its binomial has been validly published in a horticultural periodical predating any relevant taxonomic revision. However, due to the present-day scarcity and obscurity of this periodical, its proper nomenclatural evaluation has not been performed and is proposed here.

*Der Deutsche Gartenrat* was a horticultural periodical directed and edited by Andreas Voss during 1903–1906. As Voss was a friend of Otto Kuntze, known for his major but generally unaccepted reform of botanical nomenclature [70], he immediately adopted the new generic nomenclature from Kuntze’s work, including the synonymisation of *Kochia* with *Bassia* [71]. As a consequence of this generic synonymy, Voss intentionally published a new species combination, *Bassia scoparia* (L.) Voss, in a supplement to his periodical, in which he portrayed and introduced valuable ornamental plants to his audience [72]. Some authors [e.g., [73]] erroneously believed that this new combination appeared in the same periodical already in 1903, on page 289, which actually featured a review of Kuntze’s generic nomenclature without any new combinations or any specific notes on *Bassia* [74].

### 3.3. Type Variant of Bassia scoparia

Usually, *B. scoparia* is considered to have glabrous perianth segments or segments that are ciliate at the top. This variant corresponds to the type variety, *B. scoparia* var. *scoparia*.

This variety is common and has the widest distribution across temperate Eurasia and the Americas. It is widespread in the plains of Central Asia as a ruderal plant (e.g., in Xinjiang, China; pers. obs. of APS) and is often dispersed in Europe along railroad tracks.

Plants described as *Suaeda sieversiana* Pall. (BM000950576!) have prominently ciliate leaves and ciliate perianths. Such plants stand very close to the lectotype of the species name and were correctly interpreted as a subglabrous variant by Moquin-Tandon [75]. Their later interpretation as a variant with hairs in the bract axils [4] was erroneous.

### 3.4. More Pubescent Variant of Bassia scoparia

Unaided, *B. scoparia* is able to spread widely along the railroad tracks, being one of the most common railroad-adapted species in European Russia [1] and the Russian Far East ([6], as *Kochia scoparia* and *K. sieversiana*), and is also resistant to many herbicides [76]. Such plants often represent a more pubescent form of *B. scoparia* previously called *B. densiflora* or, erroneously, *B. sieversiana*. They can be characterized by hairy leaves, prominent tufts of hairs in the bract axils, and ciliate perianth.

At the rank of variety, the correct name for such more hairy plants is *B. scoparia* var. *subvillosa* (Moq.) Lambinon.

### 3.5. Halophytic Variant of Bassia scoparia

Plants with hairy axils from the saline lands of Central Siberia have long been recognized as a distinct morphotype due to their narrowly linear leaves. Originally, they were described as a variety, *Kochia scoparia* var. *angustifolia*, but, later, they were elevated to the species rank, in which they have been recently accepted as *K. angustifolia* or *Bassia angustifolia* [7,10].

The species status of this taxon was not confirmed by our phylogenetic analysis. We suggest returning to its varietal rank and provide a correct name for this variety, ***Bassia scoparia*** var. ***angustifolia*** (Turcz.) Sukhor. & Sennikov, **comb. nov.** (basionym: *Kochia scoparia* var. *angustifolia* Turcz., Bull. Soc. Imp. Naturalistes Moscou 25(4): 424 (1852)).

### 3.6. Ornamental Variant of Bassia scoparia

**Figure 4:**
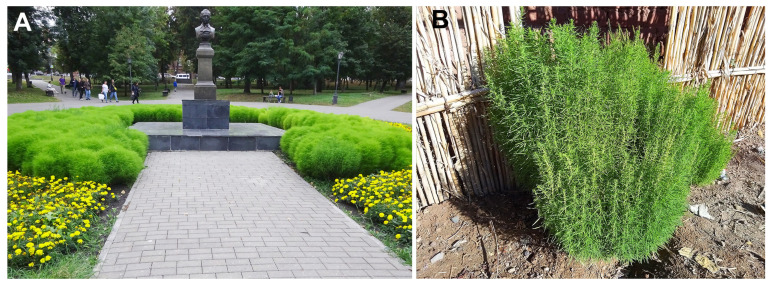
*Bassia scoparia* var. *trichophila*. (**A**) Ornamental cultivation in Penza City, Russia (August 2023, photographer A. Sukhorukov); (**B**) escaped from cultivation (Morocco, 17 December 2023, photographer J.-F. Léger).

A bushy, almost glabrous and narrow-leaved form, known as *B. scoparia* var. *trichophila* or *trichophylla*, is widely cultivated for ornamental purposes (Figure 4A) in many temperate and subtropical regions of the world [1,2,16,18,55,77,78]. Its intentional introduction is also connected with its common use as a traditional material for broom production in rural areas, e.g., in the Black Earth Region of Russia (pers. obs. of APS; [79]; see also a short note in [80]) as well as in Japan [81]. Escaped from cultivation, plants of this variety are sometimes collected in urban disturbed places, e.g., in North Africa ([73], see also Figure 4B). It seems that *B. scoparia* is an ergasiophyte (garden escape) in this region.

Despite certain morphological differences, Cinq-Mars and van den Hende [82] reported that the ornamental variety may revert to the weedy type after a few generations in the absence of nursery selection. This reversal and the variable seed quality were observed by garden practitioners already at the time of the plant’s introduction (e.g., [83,84]). Both weedy and cultivated types possess glabrous or ciliate perianth segments.

As *B. scoparia* is a promising plant for medicinal purposes [85], its broadening technical cultivation may initiate further secondary dispersal.

The nomenclature of the ornamental variety of *B. scoparia* is very intricate and has been a matter of conflicting interpretations. Seeds of this garden variety were originally collected by a farmer in the wild of Allegheny, Pennsylvania, and were eventually tried by W. Atlee Burpee, Philadelphia [86]. From this source, the seeds were received by the nursery of Haage & Schmidt, Erfurt, in the summer of 1903 [87], where new plants were produced and dried specimens were forwarded to the Kew Botanic Gardens; in return, they received an identification from Otto Stapf, who determined the plants as a new species, *Kochia trichophila* (with this exact spelling, rather than “trichophylla”). The new name was obtained by the nursery on 13 November 1903 and was quickly adopted for commercial distribution. The plants were advertised to the public in the summer of 1905 and featured in numerous notes and announcements in horticultural journals during 1905–1906.

Mabberley [88] gave priority in validation of the name *K. trichophylla* to Oskar Schmeiss, a gardener manager at Tannhof, Lindau am Bodensee, who published an information note on this ornamental plant and provided its photograph [89]. In that publication, the plants were characterized by the color of their leaves, turning from soft green to bright or blood red with autumn. Such a statement belongs to “purely aesthetic features” as defined in Art. 38.3 [90], which cannot be used in validating descriptions for new plant names. However, the characters of plant shape and foliage, when unambiguously considered diagnostic from *Kochia scoparia* (e.g., in [91]), may also serve for nomenclatural purposes. Such diagnostic statements on the morphology of these plants also appeared before 1906 (e.g., [92]); eventually, they descend from one of the contemporary trade catalogues of Haage & Schmidt, as indicated by the same plant name authorship [67]. These catalogues, not yet available on the Internet, should be searched for the place of valid publication of the name *Kochia trichophila*. The original paper catalogues are bibliographic rarities and remain inaccessible to us; for the time being, we indicate their existence for future research and provide a temporary reference to a rebuttal note by Haage & Schmidt [87], in which the conditions for valid publications were fulfilled by a reference to the diagnosis in [91] and without a reference to any previous publication of the same plant name.

The authorship of new plant names in the trade catalogues of Haage & Schmidt is a special problem that has been already discussed in the taxonomic literature [93]. The outgoing letters and publications from this nursery have always been signed by the business name, “Haage & Schmidt” (e.g., [94]), despite the fact that Johann Nicolaus Haage died long before that time and Carl Schmidt, the sole business owner in 1878–1919, concentrated on his vast enterprise management rather than on the botanical problem of naming and describing many thousands of entries in their catalogues [95]. In the absence of any ascription by the individual plant names and the unavailability of names of particular authors responsible for the potential nomenclatural novelties, we agree with the earlier suggestion [93] to ascribe the nomenclatural novelties to the business name. In order to avoid confusion with personal authorship, we propose to indicate the business authorship by plant names in quotation marks, e.g., *Kochia trichophila* Stapf ex “Haage & Schmidt”.

The name spelling of this taxon is another issue. Although many sources adopted the spelling “trichophylla” as perhaps most logical in the meaning (referring to the narrowly linear shape of the leaves), the original spelling [87] was “trichophila” (which can be translated as “hair-loving”). This spelling was maintained by Stapf [67], the original name author, thus showing that it was intentional rather than an oversight.

Möller [96] suggested the varietal rank for *Kochia trichophila* but hesitated in its acceptance. Osborn [84] was the first to definitely accept this rank with a reference to Pieters [91] and Möller [96], thus validly publishing the varietal combination.

El Mokni and Iamonico [55] cited Bailey [97] as the author of the varietal name *Bassia scoparia* var. *trichophila* (“trichophylla”). This name is absent in that book, and has never been validly published before. Although El Mokni and Iamonico [55] accepted this varietal name and provided two synonyms with full and direct references to the places of their nomenclatural publication, they failed to fulfill conditions for its valid publication unintentionally because they cited a later critical note [98] instead of the actual basionym. Herein, we supply the correct name for this ornamental plant at the rank of variety, ***Bassia scoparia*** var. ***trichophila*** (Stapf ex “Haage & Schmidt”) Sukhor. & Sennikov, **comb. nov.** (basionym: *Kochia trichophila* Stapf ex “Haage & Schmidt”, Möllers Deutsche Gärtn.-Zeitung 21(18): 219 (1906)).

Another infraspecific epithet applied to the cultivated variant of *B. scoparia* is “culta”. Notably, Graebner [99] cited its earliest publication as “*Bassia scoparia* var. *culta* Voss, Der Deutsche Gartenrat 1904 Beil. Pflanzenk. Gärtner-Neuz. 18 (1905)”, and this citation appears to be the basis for subsequent references. Our nomenclatural and bibliographic study does not confirm this citation.

This epithet first appeared in a polemic note, in which Voss [98] suggested that, should the cultivated plants of *B. scoparia* be proven stable in their morphological characters, they could have been named as *B. scoparia* f. *culta*—but, in his opinion, they have not been stable in cultivation. This means that Voss invalidly published a provisional name, which he did not accept. Ascherson’s reference to the year 1904 evidently points at the taxonomic treatment of the cultivated *Bassia scoparia* [72] rather than its infraspecific nomenclature.

The second part of Ascherson’s citation (“Beil. Pflanzenk. Gärtner-Neuz. 18 (1905)”) looks like referring to the next editorial enterprise of Voss, *Gärtner-Neuzeit*, but that periodical with the private publishing house of the same name was active later, during 1908–1912. Potentially, some privately published pamphlet (“Beilage”) could be the source of this citation, but nothing of this kind has been traced so far, and the valid publication is unlikely there because Voss was reluctant to accept the taxon. So far, we maintain the authorship of Voss tentatively for this infraspecific name, awaiting the future research in the old German horticultural literature. The citation of subsequent combinations based on the infraspecific name introduced by Voss eventually depends on the outcome of this research, although their validity is beyond doubt because they eventually refer to the description and illustration of the cultivated plants, which were provided by Voss [72]. In any case, the set of infraspecific names with the final epithet “trichophila” seems to predate the “culta” names and, therefore, the latter set has no priority in the infraspecific classification of *B. scoparia*.

### 3.7. Hirsute Variant of Bassia scoparia

**Figure 5:**
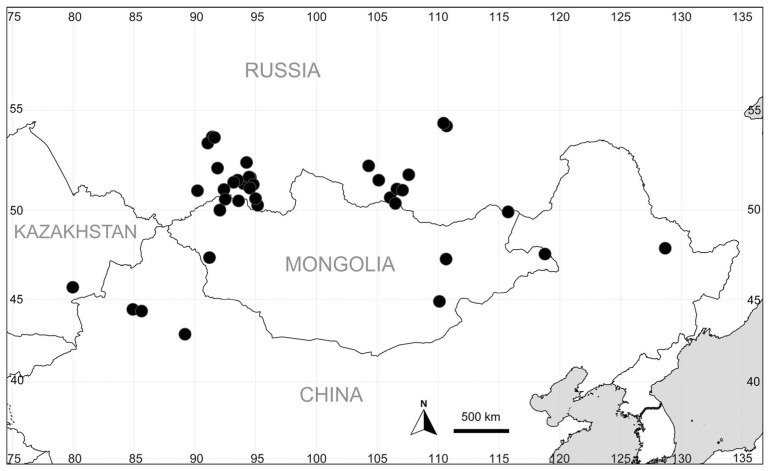
Records of *Bassia scoparia* with pubescent perianths (previously known as *Kochia albovillosa* or *K. scoparia* subsp. *hirsutissima*).

Fully pubescent perianths are present in some populations native to north-eastern China, known as *Kochia albovillosa* [9], and in mountainous Central Asia, which were described as *K. scoparia* subsp. *hirsutissima* [8]. The range of these plants encompasses East Kazakhstan, South Siberia, W, N, and NE China, and Mongolia; see also Figure 5 based on the specimens seen and Appendix A.

Previously, specimens of *B. scoparia* from South Siberia with hirsute perianths were erroneously labelled as *Kochia iranica* Bornm. ([7], specimens kept at the herbaria NS! and NSK!), now *B. odontoptera* (Schrenk) Freitag & G.Kadereit, which is clearly absent in Siberia. These misidentifications were eventually published as *B. stellaris* (Moq.) Bornm. [7], another misapplied name, and have been accepted as such in POWO [100].

Moreover, the hirsute plants were often misidentified as *B. hyssopifolia* (Pall.) Kuntze due to their shorter leaves and pubescent perianths. The latter species with uncinate (unwinged) perianth outgrowths is also absent in the mountains of South Siberia (APS, unpubl. data), and all its records from that territory belong to the more hirsute form of *B. scoparia* with glabrous or pubescent perianths.

As the other morphotypes of *B. scoparia* s.l., plants with the hairy perianths appear to be embedded in the species grade on the phylogenetic tree. This fact warrants their taxonomic inclusion in the species. The hairy morphotype can be distinguished at the rank of variety, as ***Bassia scoparia*** var. ***hirsutissima*** (Sukhor.) Sukhor. & Sennikov, **comb. nov.** (basionym: *Kochia scoparia* subsp. *hirsutissima* Sukhor., Ann. Naturhist. Mus. Wien, B 104: 700 (2003)).

Although *B. scoparia* has an extensive secondary distribution area, the hirsute plants known as *Kochia albovillosa* or *K. scoparia* subsp. *hirsutissima*, which are growing in natural habitats (sands, rocky substrates, saline soils) and are frequently found in disturbed places within their native range, have never been collected in other regions as alien plants.

## 4. Materials and Methods

### 4.1. Molecular Phylogenetic Analysis and Related Procedures

The total DNA was extracted from the leaves of the herbarium specimens using the CTAB method [101]. For the molecular phylogenetic analysis, we amplified three molecular markers: nuclear ITS [102], cpDNA loci *rpL16* intron (*rpL16* hereinafter) [103], and *atpB-rbcL* intergeneric spacer (*atpB-rbcL* hereinafter) [104]. For the amplification of the ITS, we used primers NNC–18S10 and C26A [105]. The *atpB-rbcL* locus was amplified using primers arpB-1 and rbcL1 [104]. The *rpL16* locus was amplified using primers rpL16F71 and rpL16R1516 [103]. The details of the amplification profiles are given in [104] (*atpB-rbcL*), [103] (*rpL16*), and [106] (ITS). All PCR products were sequenced on a 3730 DNA Analyzer (Applied Biosystems, Foster City, CA, USA, https://www.thermofisher.com, accessed on 11 April 2024) at the LLC Syntol, Moscow, Russia (https://www.syntol.ru, accessed on 11 April 2024) using the same primers that were used to amplify the loci. All sequences were deposited in the GenBank database (https://www.ncbi.nlm.nih.gov/genbank/, accessed on 11 April 2024); the accession numbers of the newly obtained sequences are presented in the Supplementary Material (Table S1). Sequences of each locus were aligned using the MUSCLE algorithm [107] and manually concatenated to a supermatrix for the subsequent phylogenetic analysis.

We reconstructed the phylogeny of the intensively sampled genus *Bassia* s.l. and outgroups (*Camphorosma annua* Pall., *C. lessingii* Litv., *C*. *monspeliaca* L., *C. songorica* Bunge, *Sedobassia sedoides* (Pall.) Freitag & G.Kadereit, Camphorosmoideae, Chenopodiaceae [10,108]) using a Maximum Likelihood method (ML) [109] with IQ-Tree version 1.6.12 [20], as implemented in CIPRES [110]. The partitioned phylogenetic analysis involved a concatenated matrix of all three loci listed above: ITS (85 sequences), *atpB-rbcL* (87 sequences), and *rpL16* (53 sequences). IQ-TREE [20] automatically selected the models of nucleotide substitutions for each partition based on the Bayesian information criterion (reviewed in [111]). We used the results of the “approximate likelihood-ratio test for branches” (aLRT) [21] as a measure of the clade’s support value. IQ-Tree calculated ML aLRT values following 2500 replicates.

### 4.2. Morphological Study: Taxonomy and Distributions

The specimens of *B. indica* were seen and revised in BM, BR, CHAMB, ECWP, FI, FT, G, K, LE, M, MHA, MSB, MW, W, and WU. The distribution map of *B. scoparia* with fully pubescent perianths (previously identified by APS as *Kochia scoparia* subsp. *hirsutissima*) is based on the specimens seen in LE, MHA, MW, NS, NSK, TK, and XJBI, and they were prepared using SimpleMappr online tool (http://www.simplemappr.net, accessed on 7 August 2024). All records of *B. indica* from Spain were georeferenced and further documented (also with photos) on the online observation platform observation.org (https://observation.org/species/727560/observations/?advanced=on, accessed on 4 August 2024), and these data were subsequently uploaded to GBIF (https://www.gbif.org/, accessed on 4 August 2024).

## 5. Conclusions

Despite its enormous morphological variability, *Bassia scoparia* is circumscribed here in a broader sense to achieve its monophyletic circumscription. The revised nomenclature of *B. scoparia* allows us to classify its infraspecific morphological variability at the level of variety for practical use in manuals and collections. Phylogenetically, *B. scoparia* is a sister to *B. indica*, a morphologically similar but geographically distinct species of the genus. Unlike all other *Bassia*, these two species are highly invasive, with their secondary distribution ranges overlapping, and their spread has been initiated via both unintentional and human-aided introduction pathways.

## Figures and Tables

**Table 1:** Morphological characters distinguishing *Bassia scoparia* from *B. indica*.

Character	*Bassia scoparia*	*Bassia indica*
Life form	annual, bushy or not, with lateral branches at an acute angle with the main axis	annual, bushy (tumbleweed) with horizontally spreading or deflexed lateral branches
Stem	glabrous to pubescent	pubescent
Leaves	linear to oblong, not recurved, glabrous, ciliate, or pubescent	lanceolate to oblong, often recurved, villous
Leaf axils	glabrous to pubescent	pubescent
Perianth	glabrous, ciliate, or hirsute (Central Asian and Siberian populations)	pubescent
Perianth outgrowths at fruiting stage	absent, tuberculate, or winged (plants heterodiasporic)	usually winged

## Data Availability

Data on plant distribution are contained within the article and Supplementary Material. All DNA sequences were deposited in the GenBank database (https://www.ncbi.nlm.nih.gov/genbank/); the accession numbers of the newly obtained sequences are presented in the Supplementary Material.

## Supplementary Materials

The following supporting information can be downloaded at: https://www.mdpi.com/article/10.3390/plants14030398/s1, Table S1: GenBank accession numbers of the DNA sequences (with vouchers of the newly conducted sequences). Figure S1: Phylogeny of Bassia and outgroups, recovered from the partitioned ML analysis of the concatenated dataset (ITS, atpB-rbcL intergeneric spacer, and rpL16 intron) and presented as a simplified phylogram. The scale reflects the amount of genetic change: the branch length units are nucleotide substitutions per site. The composition of the clades, the details of the obtained relationships, and the branch support values (aLRT) are given in Figure 1.

## Appendix A

Specimens of Bassia scoparia with pubescent perianths (Bassia scoparia var. hirsutissima = Kochia albovillosa).

China: Xinjiang prov., Grape Valley, Turpan, 25 June 1961, anonymous 2193 (XJBI); Xinjiang prov., Shawan county, Guan Kejian 1189 (XJBI); Xinjiang prov., Kuytun, 7 June 1979, *Guan Ke-jian 1154* (XJBI));

Kazakhstan: East Kazakhstan, Lepsy River basin, Baskan-kul Lake, 30 June 1934, I.A. Linczevsky & O.A. Linczevsky 263 (LE).

Mongolia: near Ulangom town, 23 June 1916, M. Neiburg s.n. (TK); Khovd aimak, Bulgan, 27 July 1975, O. Zhurba 947 (MW0176423); Ubsunur aimak, 12 km N of Tes vill., 26 August 1979, I. Gubanov 8106 (MW0176419);

Russia:

Buryatia Rep.: [Kurumkan distr.] Argada River, 1911, M. Korotky & P. Nikolaev 925 (MW0058705); Kurumkan distr., nr Mogoito vill, 6 August 1947, L. Sergievskaya s.n. (TK); Djidinsky distr., nr Dyrstuy vill., 1 August 1948, L. Sergievskaya & S. Gudoshnikov s.n. (TK); nr Ulan-Ude town, 14 August 1948, L. Sergievskaya s.n. (TK); Selenginsky distr., Novoselenginsk vill., 28 June 1965, Peshkova & Tarasova 1508 (NSK, TK); Kyakhta town, 28 July 1965, Peshkva & Skudenkova 2601 (TK); [Djidinsky distr.] Bulun-Ichetui vill., 10 September 1965, G. Peshkova s.n. (NSK); [Mukhorshibir’ distr.] between Ust’-Altasha & Podlopatki vill., 3 August 2016, N. Gamova s.n. (MW);

Irkutsk prov.: nr Irkutsk city, 1828, Kruse s.n. (LE) sub *Bassia hyssopifolia*;

Khakassiya Rep.: nr Abakan, Jul [without year, probably late 19th century], Klemens & Pisarev 613 (MW0058717); Altaisky distr., nr Arshanovo vill., 15 August 1968, N. Alekseeva & V. Krotova s.n. (NSK);

Krasnoyarsky Krai: Selivanikha vill. [nr Minusinsk town], August 1884, N. Martyanov 988 (LE);

Tyva Rep.: mouth of Elegest River, 5 August 1909, B. Shishkin s.n. (TK); confluence Bei-Khem & Khak-Khem Rivers, 4 August 1915, G. Miklashevskaya s.n. (LE); nr confluence of Baryk River to Enisey River, 18 July 1946, A. Shreter 297 & 801 (MHA0371561, MW0058719); Kaa-Khemsky distr., 13 July 1951, V. Skalon s.n. (NSK); Piy-Khemsky distr., Khadun River basin, 2 August 1971, E. Penkovskaya & V. Chirkova s.n. (NSK); Ulug-Khemsky distr., Senek River basin, 19 July 1972, M. Lomonosova & A. Krasnikov 3235 (MW0058698); Erzin distr., nr Erzin vill., 1270 m a.s.l., 22 July 1972, S. Timokhina & Yu. Mrykhin 2584 (MW0058718); Tes-Khemsky distr., Tannu-Ola Range, 1200 m a.s.l., 3 August 1972, V. Khanminchun & al. 100418 (MHA0371650); nr Kyzyl town, 7 July 1975, I. Krasnoborov 669 (MHA0371651, MW0058695); Kyzyl distr., nr Tselinny vill., 9 July 1975, E. Korotkova & O. Ivanova 1762 (LE, MHA0371567, MW0058714); Bai-Taiginsky distr., nr Teeli vill., 16 July 1976, S. Timokhina & S. Kochergin 363 (LE, MW0058716); Tandinsky distr., Khadyn River valley, nr Sosnovka vill., steppe with *Agropyron*, 28 July 1978, I. Neyfeld & T. Maltseva s.n. (LE, MW0058715, NS); Tes-Khemsky distr., 15 km SW of Samagaltai vill., 1140 m a.s.l., 6 August 2002, V. Nikitin & al. 705 (LE);

Zabaikalsky Krai: Borzya distr., 10 km N of Solovyovka vill., 27 July 2008, V. Chepinoga & al. 24891 (NSK).

## Appendix B

Records of *Bassia indica* from the Mediterranean Region based on the herbarium specimens seen and identifiable images from GBIF [80] and iNaturalist [81].

Algeria: El Atteuf, 2.39911 N, 3.75995 E, October 2018, Abdelwahab Chedad, https://www.inaturalist.org/observations/17748277, accessed on 13 May 2024; M’sila Region, 35.77418 N, 4.453743 E, 2021, Khellaf Rebbas https://www.gbif.org/ru/occurrence/3923317461, accessed on 13 May 2024; Biskra Region, Ain Naga, 34.72169 N, 6.24373 E, 2 December 2023, image of Abdenour Kheloufi, https://www.inaturalist.org/observations/192937440, accessed on 13 May 2024.

Cyprus: 7 km SW of Larnaka, 17 June 1996, J. Walter 7816 (W); Lefkosia, Chilonos street near Pediaios River, 155 m a.s.l., November 2002, R. Hand 3747 (B); Orounta, c. 500 m S of the village at road, 300 m a.s.l., 5 November 2002, R. Hand 3750 (B); nr Larnaca, 17 April 2006, A. Seregin & I. Privalova 650 (MW0736945); Larnaca Bay, Dhekelia, 6 November 2006, A. Sukhorukov s.n. (MW0736943, MW0736947);

Egypt: Alexandria, September 1930, E. Gauba 96 (W) as *Kochia scoparia*; Desert Institute Garden, Heliopolis, cultivated here with great success for salt reclamation, 21 October 1958, F. Hussein & V. Täckholm s.n. (LE); Cairo, 14 February 1959, sandy flat, L.E. Lodin 1051 (LE); Dumyāţ prov., El Adliya, 10 March 1981, A. El Bakry 2501 (M); [Ismailia Governorate] Ismailia town, 14 April 1984, Alaa & al. (M); [Beheira Governorate] Idku town, 25 September 1987, A.G. Fahmy 693 (M); Wadi el Gedid, nr Bashandi, 25 February 1997, J. Walter 1 & 2 (W); Farafra, 26 November 2008, B. Gianangeli 2 (FT);

Israel: Yaffa, September/October 1919, F. Meyers & J.E. Dinsmore 3865 (JE), lectotype of *Bassia joppensis*; Negev, Be’er Sheva, roadsides, 1 November 1951, J. D’Angelis 416 (AMD23736, HUJ, LE, W, WAG1448006); Be’er Sheva city, roadsides, 1 February 2011, A. Sukhorukov, M. Kushunina & E. Korolkova s.n. (MW, W); Ashkelon, 7 November 2014, A. Sukhorukov s.n. (MW0736942);

Libya: see records cited in [36], no herbarium specimens were seen by the authors;

Morocco: Essaouira towards Marrakech, ca. 30 km west of Chichaoua, gravelly roadside, 12 June 2012, *F. Verloove* 9955 (BR0000028497011: https://www.botanicalcollections.be/specimen/BR0000028497011, accessed on 5 May 2024); Agadir, Boulevard Mohammed V, 22 June 2012, *F. Verloove* 9545 (BR0000028497042: https://www.botanicalcollections.be/specimen/BR0000028497042, accessed on 5 May 2024); Jerada, (34.22549/−2.42821), 779 m a.s.l., 13 October 2014, M. Chambouleyron s.n. (ECWP); Chorfa M’daghra, Meski, (31.87000/−4.25000), 980 m a.s.l., 20 October 2019, C. Lemmel s.n. (ECWP); Boudnib, (31.94000/−3.58000), 900 m a.s.l., 22 October 2019, C. Lemmel s.n. (ECWP); Tendite, (33.66779/−3.61001), 680 m a.s.l., 29 October 2019, M. Chambouleyron s.n. (CHAMB); Errachidia, (31.98091/−4.46015), 1040 m a.s.l., 30 October 2019, M. Chambouleyron s.n. (CHAMB); Boudnid, Tazouzart, (32.07645/−3.78536), 1060 m a.s.l., 30 October 2019, M. Chambouleyron s.n. (CHAMB); Melg El Ouidane, (34.54423/−3.02484), 210 m a.s.l., 01 November 2019, M. Chambouleyron s.n. (CHAMB); Aïn Bni Mathar, (34.03349/−2.03503), 920 m a.s.l., 07 November 2019, M. Chambouleyron s.n. (ECWP); Mediouna, (33.39333/−7.53528), 190 m a.s.l., 02 October 2020, A. Tanji s.n. (ECWP); Settat, (33.07639/−7.62667), 260 m a.s.l., 04 October 2020, A. Tanji s.n. (ECWP); Berrechid, (33.23444/−7.58889), 220 m a.s.l., 04 October 2020, A. Tanji s.n. (ECWP); Berrechid, (33.27639/−7.51194), 230 m a.s.l., 04 October 2020, A. Tanji s.n. (ECWP); Marrakech, (31.69028/−7.99389), 400 m a.s.l., 17 October 2020, A. Tanji s.n. (ECWP); Marrakech, (31.71278/−7.98306), 410 m a.s.l., 17 October 2020, A. Tanji s.n. (ECWP); Kettara, (31.91944/−8.19333), 490 m a.s.l., 17 October 2020, A. Tanji s.n. (ECWP); Kettara, (31.96139/−8.25806), 530 m a.s.l., 17 October 2020, A. Tanji s.n. (ECWP); Benguérir, (32.05667/−7.95083), 430 m a.s.l., 17 October 2020, A. Tanji s.n. (ECWP); Chemmaia, Lac Zima, (32.07333/−8.64917), 360 m a.s.l., 17 October 2020, A. Tanji s.n. (ECWP); Benguérir, (32.22528/−7.95694), 460 m a.s.l., 17 October 2020, A. Tanji s.n. (ECWP); Benguérir, (32.28611/−7.95444), 510 m a.s.l., 17 October 2020, A. Tanji s.n. (ECWP); Oulad Frej, (33.00972/−8.34472), 140 m a.s.l., 20 October 2020, A. Tanji s.n. (ECWP); Moulay Abdellah, Jorf Lasfar (33.13333/−8.61028), 50 m a.s.l., 20 October 2020, A. Tanji s.n. (ECWP); Safi, (32.20861/−9.25167), 20 m a.s.l., 21 October 2020, A. Tanji s.n. (ECWP); Ounagha, (31.53778/−9.48528), 270 m a.s.l., 22 October 2020, A. Tanji s.n. (ECWP); Tlet El Henchane, (31.54306/−9.38833), 380 m a.s.l., 22 October 2020, A. Tanji s.n. (ECWP); Chichaoua, (31.56000/−8.87722), 370 m a.s.l., 22 October 2020, A. Tanji s.n. (ECWP); Sidi L’mokhtar, (31.57556/−8.97556), 390 m a.s.l., 22 October 2020, A. Tanji s.n. (ECWP); Aïn Beïda, (31.58000/−8.52583), 380 m a.s.l., 22 October 2020, A. Tanji s.n. (ECWP); Sidi L’mokhtar, (31.58417/−9.25167), 410 m a.s.l., 22 October 2020, A. Tanji s.n. (ECWP); Sidi L’mokhtar, (31.59417/−9.14222), 370 m a.s.l., 22 October 2020, A. Tanji s.n. (ECWP); Marrakech, (31.70139/−8.05389), 380 m a.s.l., 22 October 2020, A. Tanji s.n. (ECWP); Tendite, Zarzaya, (33.74749/−3.50507), m a.s.l., 09 November /2020, M. Khyi s.n. (ECWP); Oued Zem, (32.87056/−6.59250), 850 m a.s.l., 11 November 2020, A. Tanji s.n. (ECWP); Khouribga, (32.90194/−6.83639), 830 m a.s.l., 11 November 2020, A. Tanji s.n. (ECWP); Benguérir, (32.20139/−7.79611), 460 m a.s.l., 13 November 2020, A. Tanji s.n. (ECWP); Skhour Rhamna, (32.48167/−7.54750), 320 m a.s.l., 13 November 2020, A. Tanji s.n. (ECWP); El Borouj, (32.48278/−7.30611), 340 m a.s.l., 13 November 2020, A. Tanji s.n. (ECWP); El Brouj, (32.61111/−7.29472), 470 m a.s.l., 13 November 2020, A. Tanji s.n. (ECWP); El Borouj, (32.64278/−7.37806), 540 m a.s.l., 13 November 2020, A. Tanji s.n. (ECWP); Guisser, (32.66194/−7.40583), 530 m a.s.l., 13 November 2020, A. Tanji s.n. (ECWP); Guisser, (32.75778/−7.49333), 480 m a.s.l., 13 November 2020, A. Tanji s.n. (ECWP); Guisser, (32.78139/−7.51278), 520 m a.s.l., 13 November 2020, A. Tanji s.n. (ECWP); Benguérir, (32.17167/−7.66528), 420 m a.s.l., 18 November 2020, A. Tanji s.n. (ECWP); Enjil, (33.04041/−4.78275), 1610 m a.s.l., 20 September 2021, M. Chambouleyron s.n. (ECWP Figuig, (32.10301/−1.24825), 879 m a.s.l., 22 October 2021, S. Touil s.n. (ECWP); Taroudant, (30.50520/−8.71884), 310 m a.s.l., 18 November 2021, M. Chambouleyron & A. Sukhorukov 52 (ECWP); Ait Hadi, (31.41239/−8.81007), 28 December 2021, J.-F. Léger s.n. (ECWP); Fritissa, (33.55649/−3.62628), 680 m a.s.l., 08 November 2022, M. Chambouleyron & P. Herment s.n. (ECWP); Figuig, (32.10400/−1.22258), 867 m a.s.l., 10 November 2022, P. Herment & B. El Bachra s.n. (ECWP); Guercif Nord, (34.43543/−3.34575), 393 m a.s.l., 14 November 2022, P. Herment & B. El Bachra s.n. (ECWP); Saka, Al Hadadcha, (34.71607/−3.37429), 578 m a.s.l., 15 November 2022, P. Herment & B. El Bachra s.n. (ECWP); Midar, (34.96140/−3.51178), 362 m a.s.l., 15 November 2022, P. Herment & B. El Bachra s.n. (ECWP); Driouch, (34.97489/−3.43835), 314 m a.s.l., 15 November 2022, P. Herment & B. El Bachra s.n. (ECWP); El-Kebdani, (35.10662/−3.34250), 271 m a.s.l., 16 November 2022, P. Herment & B. El Bachra s.n. (ECWP); Angad, (34.80342/−1.95743), 463 m a.s.l., 17 November 2022, P. Herment & B. El Bachra s.n. (ECWP); El Aïoun, (34.59913/−2.48942), 598 m a.s.l., 18 November 2022, P. Herment & B. El Bachra s.n. (ECWP); Errachidia, (31.88371/−4.35559), 1012 m a.s.l., 22 November 2022, P. Herment & B. El Bachra s.n. (ECWP); Tinejdad, Ksar Lbour, (31.44011/−5.17371), 1065 m a.s.l., 23 November 2022, P. Herment & B. El Bachra s.n. (ECWP); Tinejdad, (31.52546/−5.03148), 1014 m a.s.l., 23 November 2022, P. Herment & B. El Bachra s.n. (ECWP); Tineghir, (31.5285/−5.52899), 1300 m a.s.l., 23 November 2022, P. Herment & B. El Bachra s.n. (ECWP); M’semrir, (31.62115/−5.85154), 1839 m a.s.l., 25 November 2022, P. Herment & B. El Bachra s.n. (ECWP); Marrakech, Rue El lkaa, (31.62654/−8.03587), 28 December 2022, J.-F. Léger s.n. (ECWP); El Jadida, (33.13874/−8.60908), 30 December 2022, J.-F. Léger s.n. (ECWP); Casablanca, Ben Abid, (33.49903/−7.86519), 30 December 2022, J.-F. Léger s.n. (ECWP); Aït Melloul, (30.32349/−9.48227), 14 December 2023, J.-F. Léger s.n. (ECWP); Al Ouidane, (31.57863/−7.78718), 17 December 2023, J.-F. Léger s.n. (ECWP); Sidi Rahal (31.64907/−7.48246), 17 December 2023, J.-F. Léger s.n. (ECWP); Tamallalt, Douar Chaoui, (31.81239/−7.50079), 17 December 2023, J.-F. Léger s.n. (ECWP); Ouarzazate (30.97537/−6.74502), 1 December 2024, M. Chambouleyron & A. Sukhorukov s.n. (field observation).

Syria: Raqqa [Ar-Raqqah], 22 October 1953, H. Pabot (G) as *Kochia scoparia*;

Spain (new records): Granada prov., Salobreña, 19 September 2022, J.M. Sola (observations and images available through https://www.inaturalist.org/observations/136038060, accessed on 25 August 2924; Castellón prov.: Villarreal, Camí del Cabeçol next to Riu Anna, bare, steppe-like area, very common, 29 August 2023 (flowers observed on 11 September 2023), *F. Verloove* 14892 (BR0000027058565V); Burriana, Avinguda de l’Unió Europea, rough ground in residential area, roadsides, etc., common, 30 August 2023, *F. Verloove* 14897 (BR0000027058480V); https://www.inaturalist.org/observations/136038060, accessed on 25 August 2024; Pilar de la Horadada, 37.871999 N, −0.799751 W, 30 November 2023, Reinier W. Akkermans https://www.gbif.org/ru/occurrence/4881137340, accessed on 25 August 2024.

Tunisia: Medenine Governorate: île de Djerba, Seguia strand, 5 January 2005, J. Lambinon 32 (BR0000005027378); [Medenine Province], Boughrara District, 20–21 m a.s.l., 17 December 2016, R. El Mokni s.n. (Herb. El Mokni); El Kantara District, 47–48 m a.s.l., 17 December 2016, R. El Mokni s.n. (Herb. Univ. Monastir); [Gabes Province], Bouchemma District, 42–43 m a.s.l., 19 December 2016, R. El Mokni s.n. (Herb. Univ. Monastir); [Sfax Province], Mahres District, 2 m a.s.l., 19 December 2016, R. El Mokni s.n. (Herb. Univ. Monastir); [Monastir Province], Aéroport International District, 6 m a.s.l., 20 April 2017, R. El Mokni s.n. (Herb. Univ. Monastir, K); [Monastir Province], Rond-point Université District, 17 m a.s.l., 5 February 2017, R. El Mokni s.n. (Herb. Univ. Monastir, MW); 23 Janvier District, 9 m a.s.l., 18 August 2017, R. El Mokni s.n. (Herb. Univ. Monastir); [Sousse Province], Bouficha District, 5–6 m a.s.l., 9 December 2015, R. El Mokni s.n. (Herb. Univ. Monastir); [Nabeul Province], Cap-Bon, Hammamet-Sud District, 7 m a.s.l., 9 December 2015, R. El Mokni s.n. (Herb. Univ. Monastir); [Bizerta Province], Kalaate Al Andalous district, 1–2 m a.s.l., 19 October 2016, R. El Mokni s.n. (Herb. Univ. Monastir).

## References

[1] Sukhorukov A.P. (2014). The Carpology of the Chenopodiaceae with Reference to the Phylogeny, Systematics and Diagnostics of Its Representatives.

[2] Aellen P. (1954). Ergebnisse einer Botanisch-Zoologischen Sammelreise durch Iran. Botanische Ergebnisse IV: Chenopodiaceae: Kochia. Mitt. Basler Bot. Ges..

[3] Benson K.M. (1955). Phenotypic Variations of *Kochia Scoparia*. Master’s Thesis.

[4] Iljin M.M., Shishkin B.K. (1936). Chenopodiaceae. Flora of USSR.

[5] Peschkova G.A. (1972). Stepnaya Flora Baikalskoy Sibiri [The Steppe Flora of the Baikal Siberia].

[6] Ignatov M.S., Kharkevich S.S. (1988). Chenopodiaceae. Sosudistye Rasteniya Sovetskogo Dal’nego Vostoka.

[7] Lomonosova M.N., Krasnoborov I.M., Malyshev L.I. (1992). Chenopodiaceae. Flora Sibiri.

[8] Mavrodiev E.V., Sukhorukov A.P. (2003). Systematische Beiträge zur Flora von Kasachstan. Ann. Naturhistorischen Mus. Wien.

[9] Nakai T., Honda M., Satake Y., Kitagawa M. (1936). Report of the First Scientific Expedition to Manchoukuo Under the Leadership of Shigeyasu Tokunago, June–October 1933. Section IV.

[10] Kadereit G., Freitag H. (2011). Molecular Phylogeny of Camphorosmeae (Camphorosmoideae, Chenopodiaceae): Implications for Biogeography, Evolution of C4-photosynthesis and Taxonomy. Taxon.

[11] Beck-Mannagetta G., Lerchenau (1909). Icones Florae Germanicae et Helvetiacae Simul Terrarum Adjacentium Ergo Mediae Europae.

[12] Scott A.J. (1978). A Revision of the Camphorosmioideae (Chenopodiaceae). Feddes Repert..

[13] Weber W.A. (1989). New Names and Combinations, Principally in the Rocky Mountain Flora—VII. Phytologia.

[14] Sukhorukov A.P., Kushunina M.A. (2020). Morphology, Nomenclature and Distribution of *Bassia monticola* (Chenopodiaceae-Amaranthaceae), a poorly known species from Western Asia. Novit. Syst. Plant Vasc..

[15] Freitag H., Kadereit G. (2014). C3 and C4 Leaf Anatomy Types in Camphorosmeae (Camphorosmoideae, Chenopodiaceae). Plant Syst. Evol..

[16] Blackwell W.H., Baechle M.D., Williamson G. (1978). Synopsis of *Kochia* (Chenopodiaceae) in North America. Sida.

[17] Turki Z., El-Shayeb F., Shehata F. (2006). Taxonomic Studies in the Camphorosmeae (Chenopodiaceae) in Egypt. 1. Subtribe Kochiinae (*Bassia*, *Kochia* and *Chenolea*). Fl. Medit..

[18] Friesen L.F., Beckie H.J., Warwick S.I., van Acker R.C. (2009). The Biology of Canadian Weeds. 138. *Kochia scoparia* (L.) Schrad. Can. J. Plant Sci..

[19] Peschkova G.A., Malyshev L.I., Peschkova G.A. (1979). Chenopodiaceae. Flora Tsentral’noy Sibiri.

[20] Minh B.Q., Schmidt H.A., Chernomor O., Schrempf D., Woodhams M.D., von Haeseler A., Lanfear R. (2020). IQ-TREE 2: New Models and Efficient Methods for Phylogenetic Inference in the Genomic Era. Mol. Biol. Evol..

[21] Anisimova M., Gascuel O. (2006). Approximate Likelihood-Ratio Test for Branches: A Fast, Accurate, and Powerful Alternative. Syst. Biol..

[22] Kitagawa M. (1939). Lineamenta Florae Manshuricae or, an Enumeration of All the Indigenous Vascular Plants Hitherto Known from Manchurian Empire Together with Their Synonymy, Distribution and Utility.

[23] Aellen P. (1941). Über einige *Kochia*-Formen aus Argentinien. Darwiniana.

[24] De Bolòs O., Vigo J. (1974). Notes sobre Taxonomia i Nomenclatura de Plantes, I. Butlletí La Inst. Catalana D’història Nat..

[25] Sukhorukov A.P., Aellen P., Edmondson J.R., Townsend C.C., Ghazanfar S.A., Edmondson J.R. (2016). Chenopodiaceae Vent. Flora of Iraq.

[26] Wight R. (1852). Icones Plantarum Indiae Orientalis: Or Figures of Indian Plants.

[27] Sukhorukov A.P., Liu P.-L., Kushunina M. (2019). Taxonomic Revision of Chenopodiaceae in Himalaya and Tibet. PhytoKeys.

[28] Hedge I., Rechinger K.H. (1997). Kochia. Flora Iranica.

[29] Heller D., Heyn C.C. (1994). Conspectus Florae Orientalis.

[30] Miller A.G., Cope T.A. (1996). Flora of the Arabian Peninsula and Socotra.

[31] Germishuizen G., Meyer N.L. (2003). Plants of Southern Africa: An Annotated Checklist.

[32] Sukhorukov A.P., Kushunina M., El Mokni R., Sáez Goñalons L., El Aouni M.H., Daniel T.F. (2018). Chorological and Taxonomic Notes on African Plants, 3. Bot. Lett..

[33] Odorico D., Nicosia E., Datizua C., Langa C., Raiva R., Souane J., Nhalungo S., Banze A., Caetano B., Nhauando V. (2022). An Updated Checklist of Mozambique’s Vascular Plants. PhytoKeys.

[34] El Ghazali G.E.B. (2020). An Annotated Checklist to the Chenopod Flora of Sudan. Int. J. Sci..

[35] Uotila P. Chenopodiaceae (pro parte majore). In Euro+Med Plantbase—The Information Resource for Euro-Mediterranean Plant Diversity. http://www.europlusmed.org.

[36] Jafri S.M.H., Rateeb F.B., Jafri S.M.H., El-Gadi A. (1978). Chenopodiaceae. Flora of Libya.

[37] Boulos L. (1995). Flora of Egypt. Checklist.

[38] Zohary M. (1966). Flora Palaestina.

[39] Hand R. (2003). Supplementary Notes to the Flora of Cyprus III. Willdenowia.

[40] Taifour H., El-Oqlah A. (2017). The Plants of Jordan. An Annotated Checklist.

[41] Draz O. (1954). Some Desert Plant and Their Uses in Animal Feeding.

[42] Bornmüller J. (1921). Zwei Neue Arten aus Süd-Palästina: Centaurea calcitrapella und Bassia joppensis Bornm. et Dinsmore. Repert. Specierum Nov. Regni Veg..

[43] Eig A.A. (1945). Revision of the Chenopodiaceae of Palestine and Neighbouring Countries. Palest. J. Bot..

[44] Amer W.M., Pullaiah T., Ielmini M.R. (2021). The Worst Invasive Species to Egypt. Invasive Alien Species: Observations and Issues from Around the World.

[45] Viney D.E. (1994). An Illustrated Flora of North Cyprus.

[46] Della A., Iatrou G. (1995). New Plant Records from Cyprus. Kew Bull..

[47] Chrtek J., Slavík B. (2001). Contribution to the Flora of Cyprus. 4. Fl. Medit..

[48] Hand R. (2020). Various Noteworthy Records of Flowering Plants in Cyprus (1996–2019) and Some Status Clarifications. Cypricola.

[49] Biodiversity of Libya Electronic Resource 2022. https://biodiversity.ly.

[50] Ali Nafea E.M. (2015). Floristic Composition of the Plant Cover at Surt Region of Libya. Catrina.

[51] Mahklouf M.H., Shakman E.A., Pullaiah T., Ielmini M.R. (2021). Invasive Alien Species in Libya. Invasive Alien Species: Observations and Issues from Around the World.

[52] Le Floc’h É., Boulos L., Véla E. (2010). Catalogue Synonymique Commenté de La Flore de Tunisie.

[53] Benmeddour T., Fenni M. (2008). Biologie et Écologie de Ganida (*Kochia scoparia* (L.) Schrad): Plante Envahissante Du Périmètre de l’Ouatya, Biskra. Aridoculture.

[54] Quezel P., Santa S. (1962). Nouvelle Flore d’Algerie et Des Regions Desertiques Meridionales.

[55] El Mokni R., Iamonico D. (2019). *Bassia scoparia* (*Amaranthaceae* s.l.) and *Sesuvium portulacastrum* (Aizoaceae), Two New Naturalized Aliens to the Tunisian Flora. Fl. Medit..

[56] Zahran M.A. (1986). Forage Potentialities of *Kochia indica* and *K. scoparia* in Arid Lands with Particular Reference to Saudi Arabia. Arab. Gulf. J. Sci. Res..

[57] Jahandiez É., Maire R.C.J.E. (1934). Catalogue des Plantes du Maroc.

[58] Molero B., Montserrat M. (2006). Quenopodiáceas Nuevas o Raras para la Flora de Marruecos. Lagascalia.

[59] Chambouleyron M., Léger J.-F. (2021). Contribution à la Connaissance de la Flore du Maroc Oriental—Moitié Orientale des Monts de Debdou et Environs d’Aïn Bni Mathar. Travaux l’Institut Sci. Série Bot..

[60] Tanji A., Taleb A. (1997). New Weed Species Recently Introduced into Morocco. Weed Res..

[61] Sukhorukov A.P., Kushunina M.A., Stepanova N.Y., Kalmykova O.G., Golovanov Y.M., Sennikov A.N. (2024). Taxonomic Inventory and Distributions of Chenopodiaceae (*Amaranthaceae* s.l.) in Orenburg Region, Russia. Biodivers. Data J..

[62] Calonje M., Sennikov A.N. (2017). In the Process of Saving Plant Names from Oblivion: The Revised Nomenclature of *Ceratozamia fuscoviridis* (Zamiaceae). Taxon.

[63] Sennikov A.N. (2024). The Taxonomic Circumscription and Nomenclatural History of *Pilosella suecica* (Asteraceae): A Special Case of Grey Literature in Taxonomic Botany. Plants.

[64] Sennikov A.N. (2024). *Taraxacum stepanekii*, a Replacement Name for *Taraxacum roseolum* Kirschner & Štěpánek Non Charit., with Nomenclatural Notes on the Taxonomic Legacy of Boris S. Kharitontsev in the Digital Era. Botanica.

[65] Voss A. (1896). Vilmorin’s Blumengärtnerei.

[66] Voss A. (1905). On Kochia trichophylla. Dtsch. Gartenrat.

[67] Stapf O. (1919). *Kochia scoparia*, forma trichophila. Curtis’s Bot. Mag..

[68] Cullen J., Knees S.G., Cubey H.S. (2011). The European Garden Flora, Flowering Plants: A Manual for the Identification of Plants Cultivated in Europe, Both out-of-Doors and Under Glass.

[69] Miller P. (1735). Gardeners Dictionary.

[70] Albrecht O. (1924). Andreas Voß. Ein Nachruf für den Forscher und Reformator. Gartenwelt.

[71] Post T., Kuntze O. (1903). Lexicon Generum Phanerogamarum.

[72] Voss A. (1904). Garten-Botanik Nr. 2799. *Bassia scoparia* A. Voss. Dtsch. Gartenrat.

[73] Maire R.C.J.E. (1962). Flore de l’Afrique Du Nord.

[74] Voss A. (1903). Internationale Einheitliche Pflanzenbenennung. Dtsch. Gartenrat.

[75] Moquin-Tandon A. (1840). Chenopodiarum Monographica Enumeratio.

[76] Kumar V., Jha P., Jugulam M., Yadav R., Stahlman P.W. (2019). Herbicide-Resistant Kochia (*Bassia scoparia*) in North America: A Review. Weed Sci..

[77] Dodd J., Randall R., Spafford J.H., Dodd J., Moore J.H. (2002). Eradication of Kochia (Bassia scoparia (L.) A.J.Scott, Chenopodiaceae) in Western Australia.

[78] Brignone N.F., Denham S.S. (2021). Toward an Updated Taxonomy of the South American Chenopodiaceae I: Subfamilies Betoideae, Camphorosmoideae, and Salsoloideae. Ann. Mo. Bot. Gard..

[79] Zimdahl R.L. (1989). Weeds and Words: The Etymology of the Scientific Names of Weeds and Crops.

[80] Judd W.S., Ferguson I.K. (1999). The Genera of Chenopodiaceae in the Southeastern United States. Harv. Pap. Bot..

[81] Clemants S.E., Association F.K. (2018). Chenopodiaceae. Flora of Kanagawa.

[82] Cinq-Mars L., van den Hende R. (1969). *Kochia scoparia* (L.) Roth (Chénopodiacées) Envahit Le Québec. Agriculture.

[83] Moorman J.W. (1906). Another Ornamental *Kochia*. Gard. Chron..

[84] Osborn A. (1906). Kochia scoparia var. trichophylla. Gard. Chron..

[85] Grabowska K., Buzdygan W., Galanty A., Wróbel-Biedrawa D., Sobolewska D., Podolak I. (2023). Current Knowledge on Genus *Bassia* All.: A Comprehensive Review on Traditional Use, Phytochemistry, Pharmacological Activity, and Nonmedical Applications. Phytochem. Rev..

[86] Pieters A.J. (1906). The Kochias. Gardening.

[87] Haage & Schmidt (1906). Kochia trichophila. Möllers Dtsch. Gärtner-Ztg..

[88] Mabberley D. (1997). The Plant-Book: A Portable Dictionary of the Vascular Plants.

[89] Schmeiss O. (1906). Kochia trichophylla. Möllers Dtsch. Gärtner-Ztg..

[90] Turland N., Wiersema J., Barrie F., Greuter W., Hawksworth D., Herendeen P., Knapp S., Kusber W.-H., Li D.-Z., Marhold K. (2018). International Code of Nomenclature for Algae, Fungi, and Plants.

[91] Pieters A.J. (1906). Kochia trichophylla = K. scoparia. Möllers Dtsch. Gärtner-Ztg..

[92] (1905). Kochia trichophila. Gardening.

[93] Smith G.F., Figueiredo E., Bischofberger M., Eggli U. (2018). Nomenclature of the Nothogenus Names × *Graptophytum* Gossot, × *Graptoveria* Gossot, and × *Pachyveria* Haage & Schmidt (Crassulaceae). Bradleya.

[94] Haage & Schmidt (1904). Neuheiten von Samen Eigener Züchtung oder Einführung für 1904. Gartenflora.

[95] Schalldach I., Wimmer C.A. (2012). Die Erfurter Handelsgärtnerei Haage & Schmidt und Ihre Kataloge. Zandera.

[96] Möller L. (1906). Nachschrift der Redaktion. Möllers Deutsche Gärtn. Zeitung.

[97] Bailey L.H. (1924). Manual of Cultivated Plants.

[98] Voss A. (1905). Eine Sogenannte “Neue” Kochia trichophylla. Dtsch. Gartenrat.

[99] Graebner P., Ascherson P., Graebner P. (1919). Chenopodiineae. Synopsis der Mittereuropäischen Flora.

[100] (2024). POWO Plants of the World Online.

[101] Doyle J.J., Doyle J.L. (1987). A Rapid DNA Isolation Procedure for Small Quantities of Fresh Leaf Tissue. Phytochem. Bull..

[102] Baldwin B.G. (1992). Phylogenetic Utility of the Internal Transcribed Spacers of Nuclear Ribosomal DNA in Plants: An Example from the Compositae. Mol. Phylogenet. Evol..

[103] Shaw J., Lickey E.B., Beck J.T., Farmer S.B., Liu W., Miller J., Siripun K.C., Winder C.T., Schilling E.E., Small R.L. (2005). The Tortoise and the Hare II: Relative Utility of 21 Noncoding Chloroplast DNA Sequences for Phylogenetic Analysis. Am. J. Bot..

[104] Chiang T.-Y., Schaal B., Peng C.-I. (1998). Universal Primers for Amplification and Sequencing a Non-Coding Spacer between the atpB and rbcL Genes of Chloroplast DNA. Bot. Bull. Acad. Sin..

[105] Wen J., Zimmer E.A. (1996). Phylogeny and Biogeography of *Panax* L. (the Ginseng Genus, Araliaceae): Inferences from ITS Sequences of Nuclear Ribosomal DNA. Mol. Phylogenet. Evol..

[106] Sukhorukov A.P., Fedorova A.V., Kushunina M., Mavrodiev E.V. (2022). *Akhania*, a New Genus for *Salsola daghestanica, Caroxylon canescens* and *C. carpathum* (Salsoloideae, Chenopodiaceae, Amaranthaceae). PhytoKeys.

[107] Edgar R.C. (2004). MUSCLE: Multiple Sequence Alignment with High Accuracy and High Throughput. Nucleic Acids Res..

[108] Kadereit G., Lauterbach M., Pirie M.D., Arafeh R., Freitag H. (2014). When Do Different C4 Leaf Anatomies Indicate Independent C4 Origins? Parallel Evolution of C4 Leaf Types in Camphorosmeae (Chenopodiaceae). J. Exp. Bot..

[109] Felsenstein J. (1981). Evolutionary Trees from DNA Sequences: A Maximum Likelihood Approach. J. Mol. Evol..

[110] Miller M.A., Pfeiffer W., Schwartz T. Creating the CIPRES Science Gateway for Inference of Large Phylogenetic Trees. Proceedings of the 2010 Gateway Computing Environments Workshop (GCE).

[111] Findley D.F. (1991). Counterexamples to Parsimony and BIC. Ann. Inst. Stat. Math..

