# Supplementary figures and images for: A Revised Taxonomy of the Bassia scoparia Complex (Camphorosmoideae, Amaranthaceae s.l.) with an Updated Distribution of B. indica in the Mediterranean Region
Source: Plants (Basel). 2025 Jan 28;14(3):398. doi: 10.3390/plants14030398 (PMC11821195; doi:10.3390/plants14030398)

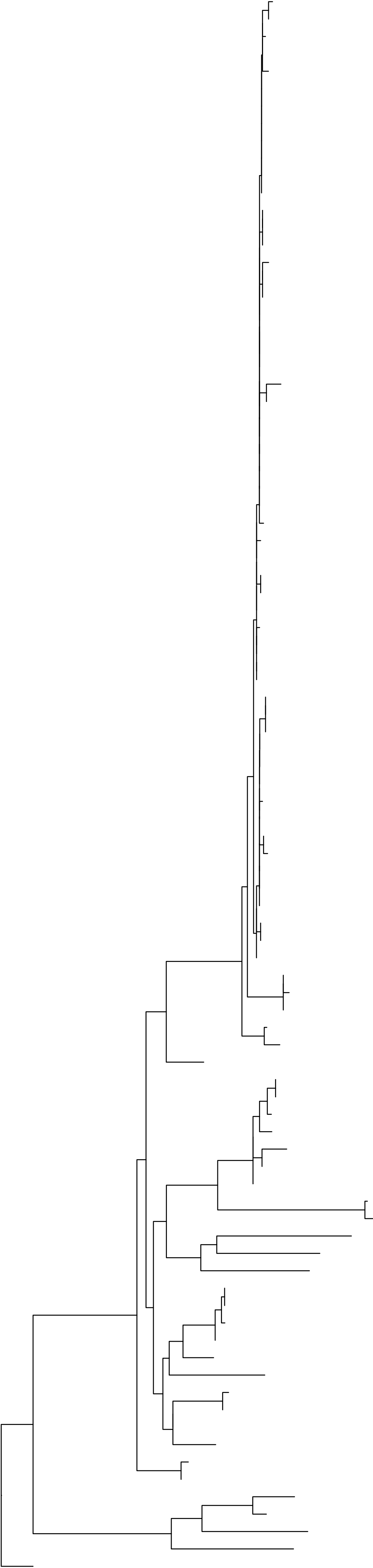

*Bassia scoparia* s.l.

*B. indica*

*B. hyssopifolia*

Outgroups

Supplement: Supplementary file 1 [file plants-14-00398-s001.zip › Figure_S1.pdf]
